# Response inhibition and exercise: from theory to translational practice

**DOI:** 10.3389/fresc.2026.1778941

**Published:** 2026-03-04

**Authors:** Daisuke Sato

**Affiliations:** 1 Institute of Health and Sport Sciences, University of Tsukuba, Tsukuba, Japan; 2 Advanced Research Initiative for Human High Performance (ARIHHP), University of Tsukuba, Tsukuba, Japan

**Keywords:** athlete, exercise, proactive inhibition, reactive inhibition, response inhibition

## Abstract

Response inhibition (RI), the ability to suppress a prepotent or ongoing action in response to sensory information, is a core control function that supports adaptive behaviour in everyday life, sport, and rehabilitation. Although RI has been widely studied in cognitive neuroscience and clinical research, reviews have rarely focused specifically on how RI relates to physical activity. This Mini Review addresses this gap by integrating three strands: (i) conceptual foundations of RI including neural mechanisms supporting proactive and reactive inhibition, (ii) elite athletes as a model of long-term adaptation, and (iii) exercise-induced modulation of RI. We first outline the distinction between proactive and reactive inhibition and summarise representative behavioural paradigms for their assessment. We then review converging evidence implicating cortico-basal ganglia circuit in stopping, highlighting candidate pathways that may differentially support anticipatory and stimulus-driven control. Building on this mechanistic framework, we discuss athlete research as a natural model for understanding experience-dependent changes in RI, and we summarise evidence that acute exercise can transiently modulate inhibitory performance, with implications for ageing and rehabilitation. By synthesising definitions, neural substrates, adaptive models, and exercise effects, we aim to advance a practical and evaluatable framework for the assessment and targeting of RI in sport, clinical rehabilitation, and daily functioning.

## 1 Inhibitory control: conceptual foundations and definition

Inhibitory control is a core component of executive function that enables suppression of inappropriate actions and irrelevant information (1, 2). Within this broad construct, response inhibition (RI), the capacity to cancel or withhold motor plans and actions, has been studied extensively because it can be operationalised with well-characterised behavioural paradigms and linked to specific neural circuits. Clinically, impaired RI is associated with impulsive and maladaptive behaviours and has been implicated across psychiatric conditions, including attention-deficit/hyperactivity disorder and several forms of addiction (3, 4).

RI is commonly conceptualised as comprising proactive and reactive inhibition (3–6). Proactive inhibition refers to anticipatory, goal-driven control that configures the motor system in advance when stopping may become necessary. This preparatory state can increase the effectiveness of subsequent stopping by adjusting response readiness and pre-engaging inhibitory pathways (5). In contrast, reactive inhibition is a stimulus-driven process triggered by an external stop signal, resulting in rapid cancellation of an ongoing or imminent action (7). Because reactive stopping must override motor commands that are already unfolding, it is often time-critical and can be vulnerable under high cognitive load or contextual uncertainty (8). Although proactive and reactive inhibition are frequently treated as separable components, they are typically considered to rely on partially shared, partially distinct mechanisms (9, 10). In sport and exercise contexts, proactive inhibition is most relevant when athletes can anticipate potential holding or/and changing actions, whereas reactive inhibition is emphasised when cancelling action must be initiated rapidly in response to unpredictable external events.

Two paradigms are widely used to index these components. In the Go/NoGo task (GNT), participants respond rapidly to Go stimuli and withhold responses to NoGo stimuli; performance is typically quantified using reaction times (RTs) on Go trials and commission errors on NoGo trials. In the stop-signal task (SST), participants perform a speeded choice response to Go stimuli but must cancel their response when an infrequent stop signal appears shortly after the Go cue. Because the stop signal is not predictable, the SST is commonly used to index reactive inhibition, with stop-signal reaction time (SSRT) providing an estimate of stopping latency (7, 11, 12). Despite extensive use, existing evidence remains insufficient to determine the degree to which GNT and SST isolate dissociable inhibitory processes (6, 13). Direct within-participant comparisons that include both tasks are therefore important for clarifying common and distinct behavioural and neural signatures of proactive and reactive inhibition (14, 15).

Clarifying these components benefits from a mechanistic account of the underlying neural circuitry. Neuroimaging and electrophysiological studies consistently implicate a cortico-basal ganglia network in stopping, with converging evidence for cortical nodes such as the right inferior frontal gyrus and the pre-supplementary motor area (16, 17). Within this network, the hyperdirect front-subthalamic pathway has been proposed to support rapid, global suppression, consistent with the time-critical demands of reactive inhibition (5, 18). Proactive inhibition, by contrast, is often described as a preparatory mode that can be configured in advance, plausibly relying more strongly on fronto-striatal-pallidal circuit that supports goal-dependent adjustments of response readiness (5). Importantly, connectivity evidence suggests that these pathways may operate in a complementary manner rather than as a strict dichotomy. Both proactive and reactive demands can modulate interactions within an IFG-SMA-STN-M1 circuit, while proactive control may additionally recruit longer front-striatal routes (18, 19). Finally, meta-analytic work indicates that basal ganglia mechanisms implicated in motor stopping overlap with those involved in suppressing unwanted memory retrieval, suggesting a broader role of basal ganglia circuit in inhibitory control across domains (9). Together, these neural accounts motivate the view that inhibitory control is shaped by experience and context, and they provide a mechanistic rationale for examining RI in athletes and in relation to physical activity.

## 2 Response inhibition in the athlete

Athletes provide a useful model for understanding response inhibition because many sports require rapid action selection under time pressure, distraction, and continuously changing situational demands, where inappropriate responses must be withheld or cancelled (20). These environments impose recurrent demands on inhibitory control in sport-specific contexts and encourage anticipatory regulation of action readiness when stopping is plausible but uncertain. This perspective aligns with the distinction between open-skill and closed-skill sports, in which open-skill athletes are assumed to face more frequent and varied inhibition demands embedded in training and competition (21, 22). Complementing laboratory paradigms, sport-relevant decision-making tasks further suggest that long-term practice in sports with strong inhibitory components can translate into better task performance and more rapid engagement of task-relevant functional processes (23). Collectively, athlete-focused research offers an informative approach for characterising how sport-specific experience shapes the mechanisms of response inhibition.

Evidence on proactive aspects of RI in athletes has often been drawn from GNT studies, but findings are mixed and depend strongly on how proactive control is operationalised. Athlete-non-athlete differences are detected more consistently in RT-related indices, including faster responding, reduced trial-to-trial variability, and distributional shifts in RT, patterns commonly interpreted as reflecting more efficient response preparation and action selection shaped by sport-specific practice (23–26). By contrast, group differences in commission errors are reported less frequently, and evidence for reliably lower false-alarm rates in athletes remains limited (27). This divergence likely reflects measurement properties. In typical GNT implementations, false-alarm rates can be low in healthy samples, limiting sensitivity to individual or group differences (28). Moreover, false alarms are strongly influenced by task parameters (for example, NoGo probability and stimulus timing) and by instruction-induced speed-accuracy settings, each of which can materially alter response tendencies and error rates (29–32). Notably, several studies report robust electrophysiological differences, such as earlier and or larger NoGo N2 and altered NoGo P3, even when behavioural performance is comparable, suggesting experience-dependent changes in the efficiency and temporal dynamics of inhibitory processing (20, 33, 34). Overall, the GNT literature indicates that conclusions about proactive inhibition in athletes hinge on construct definition and measurement choice, underscoring the value of integrating behavioural and neurophysiological markers.

Reactive inhibition has been examined extensively using the SST, with SSRT providing a well-established behavioural index. Meta-analytic evidence indicates that athletes show shorter SSRTs than non-athletes, with a small but statistically reliable overall effect corresponding to an estimated mean difference of approximately 17 ms (35). This benefit appears more robust in externally paced, cognitively demanding sports, where athletes must repeatedly cancel or revise actions under severe time constraints and rapidly changing perceptual-motor demands (36). Consistent with this account, tennis players (open-skill) have been reported to exhibit shorter SSRTs than swimmers (closed-skill) and sedentary controls, whereas swimmers did not differ from controls (36). However, sport type is not a deterministic moderator. Both tennis players and swimmers have also been shown to outperform non-athletes with no reliable difference between athlete groups, suggesting that reactive stopping can be enhanced in some closed-skill cohorts depending on training history and sample characteristics (21). Null findings have nevertheless been reported, including no SSRT differences among taekwondo athletes, swimmers, and controls (37) and no SSRT differences between endurance winter-sport athletes and non-athletes (38). Importantly, our study supports the possibility of more generalisable enhancements in reactive stopping by showing shorter SSRTs in kendo athletes than non-athletes across visual, auditory, and somatosensory stop signals (39). Beyond group means, SSRT has been linked to sport performance indicators such as self-reports and coaches' ratings, particularly at higher expertise levels, positioning reactive inhibition as a plausible, though modest, candidate marker of athletic expertise (35, 40). Interpretation should also consider SST implementation because strategic response slowing and feedback contingencies can influence stopping performance and may contribute to heterogeneity across studies (21). These findings motivate the complementary question of whether inhibitory control can also be modulated acutely by exercise bouts or targeted interventions, beyond longer-term adaptations associated with sport expertise.

## 3 Effects of exercise interventions on response inhibition

Acute exercise can modulate proactive inhibition indexed by the GNT, but the literature is best characterised as dose- and timing-dependent. When the GNT is administered during exercise, very high intensity can impair performance. For example, short-duration treadmill running at high intensity produced slower RTs and more errors relative to rest or moderate intensity, consistent with an arousal-fatigue trade-off (41). When inhibition is assessed after exercise, benefits are more likely to emerge under conditions of elevated executive demand or vulnerability. In college students with smartphone addiction, a single bout of aerobic exercise improved RI (42). Similarly, in heroin addicts, acute aerobic exercise was associated with improved RI alongside electrophysiological modulations, suggesting transient facilitation of control-related processing in a clinical population (43). However, behavioural indices may remain unchanged even when neurophysiological markers shift. In young adults with obesity, moderate-intensity exercise increased NoGo-N2 amplitude without clear behavioural improvement, implying facilitation of early inhibitory or conflict-monitoring processes that does not necessarily translate into overt accuracy gains (44). Relatedly, among smokers with nicotine dependence, acute yoga and aerobic exercise altered effect and neurocognitive markers (for example, P3 amplitude) with broadly comparable behavioural performance, indicating that acute exercise bouts may influence processing efficiency even when accuracy is stable (45). Overall, acute effects on proactive inhibition appear most detectable when intensity is kept within an optimal range, often moderate, and when outcomes include both behavioural and neurophysiological indices.

Evidence for acute exercise effects on reactive inhibition, operationalised as SSRT in the SST, is comparatively more convergent in showing transient improvements in stopping efficiency, particularly after moderate-to-vigorous aerobic exercise. A foundational time-course study reported that moderate-intensity cycling shortened SSRT and sped Go responding with benefits persisting for tens of minutes after the bout (46). Acute benefits have also been documented in high-demand populations. In male violent perpetrators, a single aerobic session improved inhibitory control in an emotional SST (47). In adults with ADHD, acute aerobic exercise at moderate and high intensities improved inhibitory control and was accompanied by changes in corticospinal excitability assessed by TMS, suggesting that acute exercise can influence both behavioural stopping and its physiological substrates (48). Taken together, the SST literature supports a model in which acute exercise can temporarily sharpen reactive stopping, while variability across studies is expected due to differences in intensity prescription, recovery interval, and SST implementation details that affect strategic slowing and stopping probability (46, 48).

Future work should move beyond testing whether acute exercise effects exist and instead specify which component is modulated, under what conditions, and for whom. Dose-response designs that vary intensity, duration, and modality, together with systematic manipulation of measurement timing, are needed to separate inhibitory modulation from non-specific arousal or fatigue effects (46). Because the GNT and SST are sensitive to speed-accuracy trade-offs and, in the SST, strategic slowing, studies should implement controls that minimise these confounds and report robust metrics, including distributional and variability indices for RT and model-consistent SSRT estimation (7, 30, 49). Behavioural endpoints should be complemented by neural measures (ERP, fNIRS or fMRI, TMS) to detect changes in processing efficiency when accuracy is constrained by ceiling or floor effects (30). Of these approaches, event-related electrophysiological measures such as ERPs (event-related potentials) and event-related oscillatory indices are well suited for resolving the temporal dynamics of inhibitory processing. ERP components such as N2 and P3 can index conflict monitoring and inhibitory processing, although their functional specificity depends on task demands and should be interpreted cautiously; oscillatory markers (e.g., midfrontal theta and frontal alpha/beta dynamics) provide complementary evidence about transient changes in control implementation. Recent acute-exercise studies have begun to test whether these electrophysiological indices during inhibition tasks change under specific exercise and task-demand combinations, but findings appear mixed across markers and protocols, underscoring the need for better-powered and better-controlled mechanistic designs (50, 51). Finally, incorporating moderators such as baseline fitness, sleep, and symptom burden will help explain heterogeneity and identify contexts in which acute exercise yields reliable benefits (48). Together, this approach will clarify when and why proactive vs. reactive inhibition is transiently altered by exercise and will provide a mechanistic bridge to longer-term interventions.

## 4 Translational implications

Distinguishing proactive and reactive inhibition provides a useful conceptual framework for thinking about control modes and their task expressions (5, 52). In clinical and applied settings, this distinction may also help generate hypotheses about component-specific vulnerabilities and intervention targets by linking behavioural phenotypes to partially separable underlying neural systems across disorders. Tasks that emphasise proactive control (e.g., go/no-go variants that manipulate expectancies or cue-based preparation) can be informative when strategic adjustments and anticipatory control are central, whereas stop-signal paradigms and SSRT better capture time-critical cancellation demands (49). Emerging clinical evidence suggests that these components can be differentially affected across disorders: for example, early-stage Parkinson's disease has been reported to show a more pronounced impairment in reactive stopping than in proactive control (53). Moreover, exercise-based interventions may differentially modulate inhibition components; one controlled study in Parkinson's disease reported improvements in reactive inhibition with no clear change in proactive inhibition (54). In stroke rehabilitation, short-term aerobic exercise training has also been linked to improved inhibitory control alongside changes in stimulus-evoked electrophysiological markers, supporting the plausibility of component-specific prescription strategies (55). Together with umbrella-level evidence that exercise benefits executive function across populations (56), these findings support a more explicit component-to-prescription mapping when extending RI research beyond sport performance.

## 5 Conclusion

This mini review synthesised conceptual, neural, and applied evidence linking response inhibition and physical activity. Overall, long-term sport-specific experience, particularly in cognitively demanding and externally paced sports, is associated with modest advantages in reactive inhibition, whereas evidence for proactive inhibition in athletes depends strongly on task implementation and measurement choice (35, 40). Acute exercise can also modulate RI, with more consistent behavioural benefits for reactive stopping after moderate-to-vigorous aerobic bouts and more variable effects for proactive inhibition that may be detected more sensitively when behavioural and neurophysiological outcomes are combined (46, 48). Moving forward, harmonised task implementation and multimodal measurement will be essential for identifying exercise prescriptions that optimise specific components of response inhibition in sport, rehabilitation, and healthy ageing programmes.

A suggested framework for experimental and practical implementations is as follows: (1) define whether the intended target is proactive inhibition (anticipatory control) or reactive inhibition (stopping/cancellation); (2) select tasks and metrics that align with that target and explicitly report potential speed–accuracy trade-offs; (3) when feasible, combine behavioural outcomes with neural markers to help separate true inhibitory changes from strategy; and (4) report exercise dose and the timing of testing/training relative to the exercise bout to facilitate cross-study synthesis and translation into practice.
